# Triclinic polymorph of 1-hy­droxy­cyclo­hexa­necarb­oxy­lic acid

**DOI:** 10.1107/S2414314625009794

**Published:** 2025-11-11

**Authors:** Lubabalo Ndima, Eric Cyriel Hosten, Richard Betz

**Affiliations:** a Nelson Mandela University, Summerstrand Campus, Department of Chemistry, University Way, Summerstrand, PO Box 77000, Port Elizabeth, 6031, South Africa; University of Aberdeen, United Kingdom

**Keywords:** crystal structure, polymorph

## Abstract

The asymmetric unit of the title compound, C_7_H_12_O_3_, an α-hy­droxy­carb­oxy­lic acid, contains two complete mol­ecules. In the extended structure, O—H⋯O hydrogen bonds connect the mol­ecules into sheets lying perpendicular to the crystallographic *b* axis.

## Abstract 2

The title compound is a derivative of cyclo­hexa­necarb­oxy­lic acid bearing a hydroxyl group in its α-position and is a polymorph of the previously reported monoclinic form.

## Graphical abstract

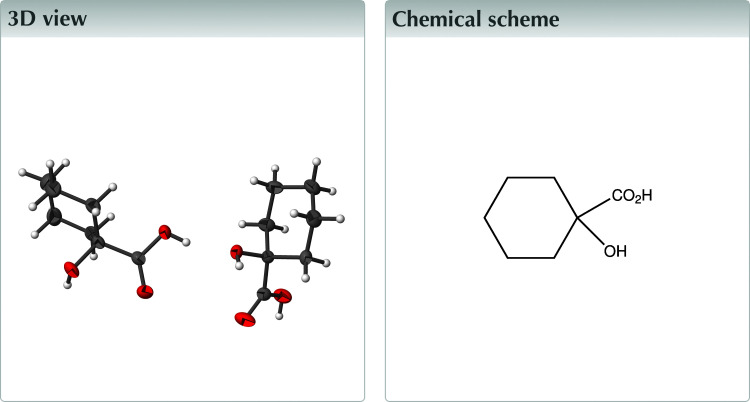

## Structure description

The Krebs cycle – also known as the citric acid cycle – is at the centre of metabolic processes in aerobic organisms. It involves a number of hy­droxy­carb­oxy­lic acids that constitute intriguing chelating ligands for a variety of transition metals of pharmaceutical inter­est (McMurry, 2008 ▸). These acids classify as potential chelating ligands which have found widespread use in coordination chemistry due to the increased stability of coordination compounds they can form in comparison to monodentate ligands (Gade, 1998 ▸). Hy­droxy­carb­oxy­lic acids are a particularly inter­esting in this aspect as they offer two functional groups that – depending on the individual requisite experimental conditions – can either act as fully neutral, fully anionic or mixed neutral-anionic donors. Upon varying the substitution pattern on the hydro­carbon backbone, the acidity of the respective hydroxyl groups can be fine-tuned over a wide range and they may, thus, serve as probes for establishing the rules in which p*K*_a_ range coordination to various central atoms can be observed. Furthermore, the steric pretence of potential substituents may give rise to unique coordination and bonding patterns. Given the multidentate nature of hy­droxy­carb­oxy­lic acids encountered in the Krebs cycle it appears prudent to investigate simpler ‘cut outs’ with a more limited number of donor sites to avoid more complex mixtures of reaction products in envisioned synthesis procedures, thus prompting the diffraction study of the title compound to allow for comparisons of metrical parameters of the free ligand and the ligand in envisioned coordination compounds. The present study confirms our continued inter­est into structural aspects of α-hy­droxy­carb­oxy­lic acids such as 1-hy­droxy­cyclo­propane­carb­oxy­lic acid (Betz & Klüfers, 2007*a* ▸), 1-hy­droxy­cyclo­butane­carb­oxy­lic acid (Betz & Klüfers, 2007*b* ▸), 1-hy­droxy­cyclo­penta­necarb­oxy­lic acid (Betz & Klüfers, 2007*c* ▸), 2-hydroxy­bi­cyclo­(2.2.1)heptane-2-*endo*-carb­oxy­lic acid (Betz & Klüfers, 2007*d* ▸), hy­droxy­isovaleric acid (Dasi *et al.*, 2024 ▸) or *tert*-butyl­glycolic acid (Betz *et al.*, 2007 ▸). Furthermore, geometrical data for glycolic acid (Ellison *et al.*, 1971 ▸; Pijper, 1971 ▸) and l-lactic acid (Schouten *et al.*, 1994 ▸; Yang *et al.*, 2021 ▸) are apparent in the literature.

The structure of a monoclinic polymorph (space group *P*2_1_/*c*) of the title compound has been reported earlier (Cambridge Structural Database refcode SIMCEX; Xu *et al.*, 2007 ▸), where the sample was recrystallized from ‘petrol (sic) ether’ solution. The very brief discussion in this paper provided an incorrect analysis of the hydrogen-bonding pattern (see below).

**Figure 1:**
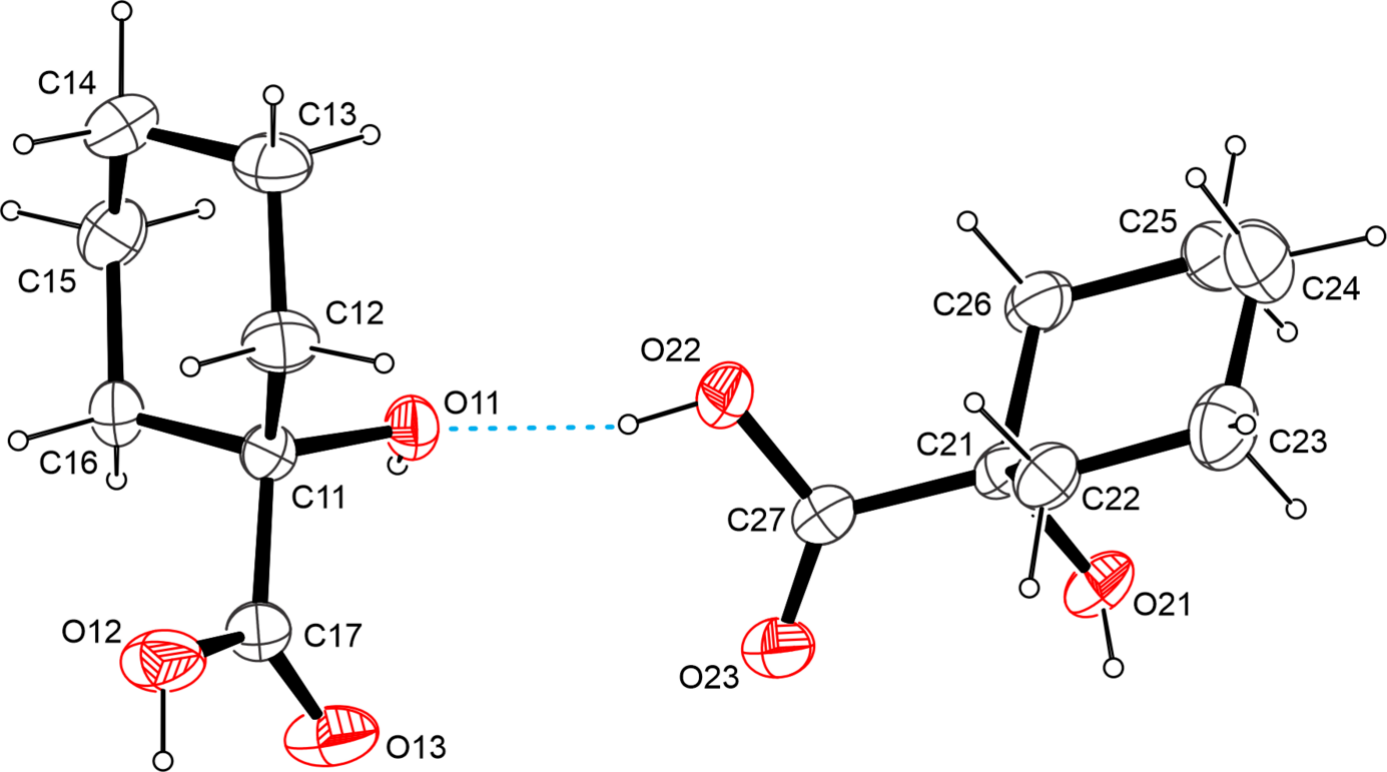
The mol­ecular structure of the title compound with displacement ellipsoids drawn at the 50% probability level.

The title compound, C_7_H_12_O_3_, is a derivative of cyclo­hexa­necarb­oxy­lic acid featuring a hy­droxy group in the α-position. The asymmetric unit contains two mol­ecules. The C=O bond lengths in the carboxyl groups are 1.3030 (13) and 1.3206 (12) Å, which are in good agreement with other carb­oxy­lic acids whose metrical parameters have been deposited with the Cambridge Structural Database (Groom *et al.*, 2016 ▸). Both six-membered rings adopt a ^1^*C*_4_ (chair) conformation (Boeyens, 1978 ▸) with the hydroxyl groups invariably occupying the axial position (Fig. 1 ▸).

**Figure 2:**
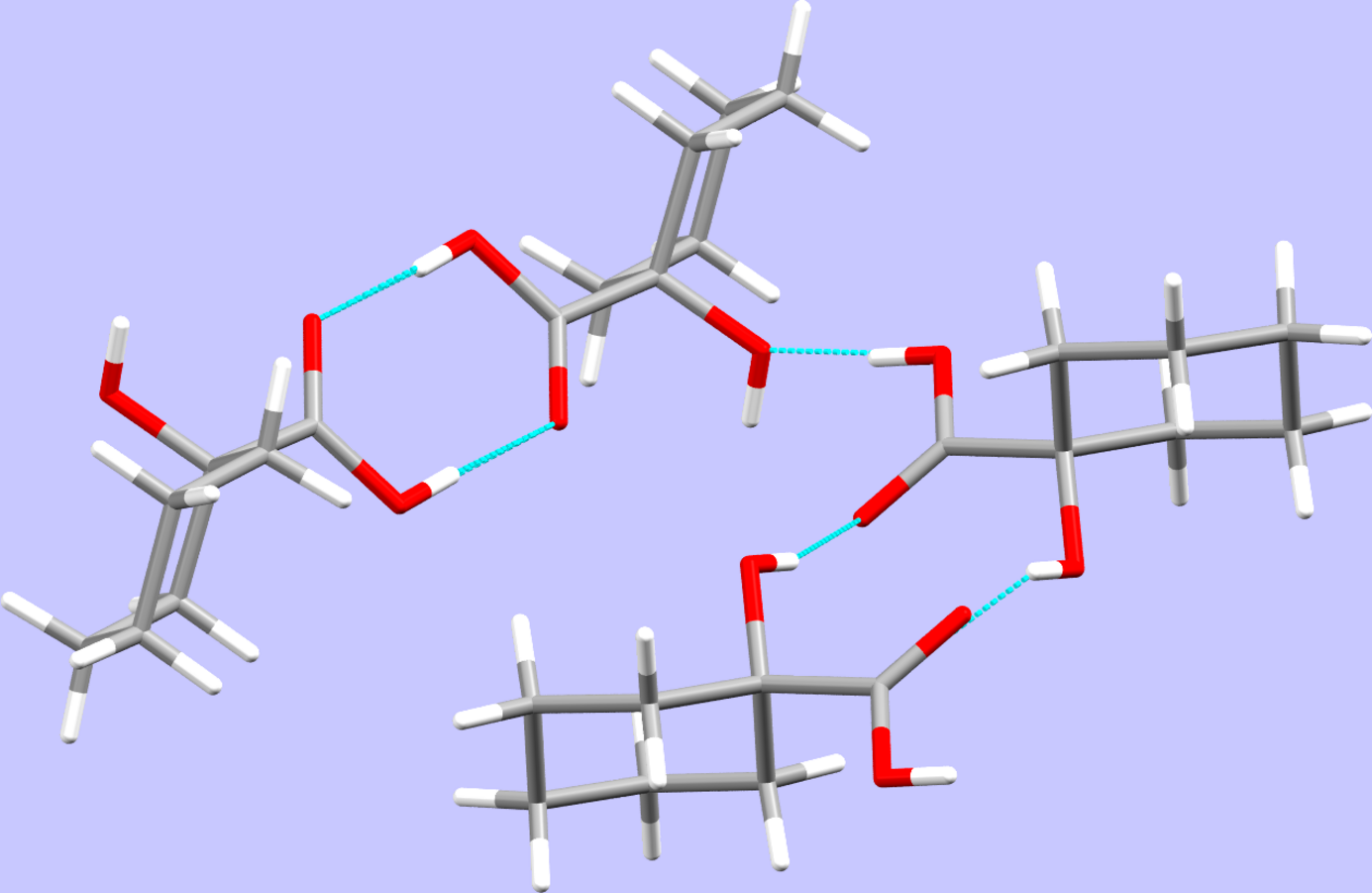
Selected inter­molecular contacts in the extended structure of the title compound, viewed along [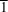
00].

In the crystal, O—H⋯O hydrogen bonds (Table 1 ▸) connect the mol­ecules into sheets lying perpendicular to the crystallographic *b* axis. The carboxyl groups in the first (C11) mol­ecule give rise to the common pattern of forming centrosymmetric dimers based on hydrogen bonding while a similar cyclic pattern is observed for the second (C21) mol­ecule present in the asymmetric unit, however, in the latter case involving the alcoholic hydroxyl group as donor and the ketone-type oxygen atom of a symmetry-generated equivalent mol­ecule as acceptor. Furthermore, the alcoholic hydroxyl group of the first mol­ecule employs the oxygen atom of the second mol­ecule’s alcoholic hy­droxy group as acceptor while the carb­oxy­lic OH group of the second mol­ecule establishes an O—H⋯O inter­action to the oxygen atom of the alcoholic hydroxyl group of the first mol­ecule, thus extending the dimeric patterns to the two-dimensional connectivity pattern as described above. In terms of graph-set analysis (Etter *et al.*, 1990 ▸), the hydrogen bonding pattern can be described as *DDR^2^_2_(8)R^2^_2_(10)* on the unary level (Fig. 2 ▸). While the hydrogen bonding pattern in the monoclinic polymorph of the title compound is stated erroneously as giving rise ‘to a hydrogen-bonded ten-membered ring’ (Xu *et al.*, 2007 ▸), the correct analysis of the hydrogen bonding in the monoclinic polymorph shows the presence of a centrosymmetric twelve-membered ring established by O—H⋯O inter­actions supported by the carboxyl group’s H atom to the oxygen atom of the alcoholic group and, in turn, the latter’s H atom seeking the ketonic oxygen atom as acceptor. The graph-set descriptor on the unary level would thus be *R^4^_4_(12)* for the monoclinic polymorph.

## Synthesis and crystallization

The compound was obtained following a standard procedure by reacting *ortho*-toluidine with KSCN and bromine in acetic acid (Becker *et al.*, 2000 ▸). Crystals suitable for the diffraction study were obtained upon free evaporation of the reaction mixture after workup at room temperature.

## Refinement

Refinement details are summarized in Table 2 ▸.

## Supplementary Material

Crystal structure: contains datablock(s) I. DOI: 10.1107/S2414314625009794/hb4541sup1.cif

Structure factors: contains datablock(s) I. DOI: 10.1107/S2414314625009794/hb4541Isup2.hkl

Supporting information file. DOI: 10.1107/S2414314625009794/hb4541Isup3.cml

CCDC reference: 2500425

Additional supporting information:  crystallographic information; 3D view; checkCIF report

## Figures and Tables

**Table 1:** Hydrogen-bond geometry (Å, °)

*D*—H⋯*A*	*D*—H	H⋯*A*	*D*⋯*A*	*D*—H⋯*A*
O11—H11⋯O21^i^	0.84	1.89	2.7195 (10)	169
O12—H12⋯O13^ii^	0.84	1.80	2.6359 (11)	176
O21—H21⋯O23^iii^	0.84	1.95	2.7716 (11)	166
O22—H22⋯O11	0.84	1.84	2.6594 (10)	164
C26—H26*A*⋯O13^i^	0.99	2.58	3.4349 (14)	145

Symmetry codes: (i) 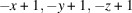; (ii) 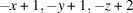; (iii) 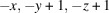.

**Table 2:** Experimental details

Crystal data	
Chemical formula	C_7_H_12_O_3_
*M*_r_	144.17
Crystal system, space group	Triclinic, *P* 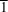
Temperature (K)	200
*a*, *b*, *c* (Å)	6.5906 (2), 11.1237 (3), 11.3502 (3)
α, β, γ (°)	109.798 (1), 96.912 (1), 102.830 (1)
*V* (Å^3^)	745.83 (4)
*Z*	4
Radiation type	Mo *K*α
μ (mm^−1^)	0.10
Crystal size (mm)	0.59 × 0.54 × 0.35

Data collection	
Diffractometer	Bruker APEXII CCD
Absorption correction	Multi-scan (*SADABS*; Krause *et al.*, 2015 ▸)
*T*_min_, *T*_max_	0.969, 1.000
No. of measured, independent and observed [*I* > 2σ(*I*)] reflections	23049, 3702, 3151
*R*_int_	0.019
(sin θ/λ)_max_ (Å^−1^)	0.668

Refinement	
*R*[*F*^2^ > 2σ(*F*^2^)], *wR*(*F*^2^), *S*	0.035, 0.093, 1.04
No. of reflections	3702
No. of parameters	186
H-atom treatment	H-atom parameters constrained
Δρ_max_, Δρ_min_ (e Å^−3^)	0.32, −0.15

Computer programs: *APEX2* and *SAINT* (Bruker, 2014 ▸), *SHELXS97* (Sheldrick 2008 ▸), *SHELXL2019/3* (Sheldrick, 2015 ▸), *ORTEP-3 for Windows* (Farrugia, 2012 ▸), *Mercury* (Macrae *et al.*, 2020 ▸), *SHELXL2019*/3 (Sheldrick, 2015 ▸) and *PLATON* (Spek, 2020 ▸).

## full crystallographic data

### 1-Hydroxycyclohexanecarboxylic acid Crystal data

**Table:**

C_7_H_12_O_3_	*Z* = 4
*M**_r_* = 144.17	*F*(000) = 312
Triclinic, *P*1	*D*_x_ = 1.284 Mg m^−^^3^
*a* = 6.5906 (2) Å	Mo *K*α radiation, λ = 0.71073 Å
*b* = 11.1237 (3) Å	Cell parameters from 9996 reflections
*c* = 11.3502 (3) Å	θ = 2.2–28.3°
α = 109.798 (1)°	µ = 0.10 mm^−^^1^
β = 96.912 (1)°	*T* = 200 K
γ = 102.830 (1)°	Block, colourless
*V* = 745.83 (4) Å^3^	0.59 × 0.54 × 0.35 mm

### 1-Hydroxycyclohexanecarboxylic acid Data collection

**Table:**

Bruker APEXII CCD diffractometer	3702 independent reflections
Radiation source: sealed tube	3151 reflections with *I* > 2σ(*I*)
Graphite monochromator	*R*_int_ = 0.019
φ and ω scans	θ_max_ = 28.3°, θ_min_ = 2.0°
Absorption correction: multi-scan (SADABS; Krause *et al.*, 2015)	*h* = −8→8
*T*_min_ = 0.969, *T*_max_ = 1.000	*k* = −14→14
23049 measured reflections	*l* = −15→15

### 1-Hydroxycyclohexanecarboxylic acid Refinement

**Table:**

Refinement on *F*^2^	Secondary atom site location: difference Fourier map
Least-squares matrix: full	Hydrogen site location: inferred from neighbouring sites
*R*[*F*^2^ > 2σ(*F*^2^)] = 0.035	H-atom parameters constrained
*wR*(*F*^2^) = 0.093	*w* = 1/[σ^2^(*F*_o_^2^) + (0.0427*P*)^2^ + 0.1705*P*] where *P* = (*F*_o_^2^ + 2*F*_c_^2^)/3
*S* = 1.04	(Δ/σ)_max_ < 0.001
3702 reflections	Δρ_max_ = 0.32 e Å^−^^3^
186 parameters	Δρ_min_ = −0.15 e Å^−^^3^
0 restraints	Extinction correction: SHELXL-2019/2 (Sheldrick 2015), Fc^*^=kFc[1+0.001xFc^2^λ^3^/sin(2θ)]^-1/4^
Primary atom site location: structure-invariant direct methods	Extinction coefficient: 0.036 (5)

### 1-Hydroxycyclohexanecarboxylic acid Special details

**Table:**

Geometry. All esds (except the esd in the dihedral angle between two l.s. planes) are estimated using the full covariance matrix. The cell esds are taken into account individually in the estimation of esds in distances, angles and torsion angles; correlations between esds in cell parameters are only used when they are defined by crystal symmetry. An approximate (isotropic) treatment of cell esds is used for estimating esds involving l.s. planes.
Refinement. The carbon-bound H atoms were placed in calculated positions (C—H = 0.99 Å) and were included in the refinement in the riding model approximation, with *U*_iso_(H) = 1.2*U*_eq_(C). The H atoms of the hydroxyl groups were allowed to rotate with a fixed angle around the C—O bond to best fit the experimental electron density (HFIX 147 in the *SHELX* program suite with *U*_iso_(H) = 1.5*U*_eq_(O).

### 1-Hydroxycyclohexanecarboxylic acid Fractional atomic coordinates and isotropic or equivalent isotropic displacement parameters (Å^2^)

**Table:**

	*x*	*y*	*z*	*U*_iso_*/*U*_eq_	
O11	0.67725 (11)	0.34334 (7)	0.65734 (6)	0.02704 (17)	
H11	0.760802	0.417523	0.669574	0.041*	
O12	0.58881 (15)	0.35087 (8)	0.96223 (8)	0.0422 (2)	
H12	0.537786	0.401756	1.015974	0.063*	
O13	0.58614 (15)	0.49905 (8)	0.87073 (8)	0.0421 (2)	
O21	0.02975 (12)	0.43479 (7)	0.32898 (8)	0.03120 (18)	
H21	−0.061970	0.454671	0.371597	0.047*	
O22	0.34101 (13)	0.27428 (7)	0.46871 (7)	0.03457 (19)	
H22	0.433087	0.304288	0.537272	0.052*	
O23	0.27903 (13)	0.47184 (8)	0.55248 (8)	0.0369 (2)	
C11	0.71553 (15)	0.31231 (9)	0.76821 (9)	0.0238 (2)	
C12	0.61015 (18)	0.16307 (10)	0.72850 (11)	0.0326 (2)	
H12A	0.616442	0.140314	0.805712	0.039*	
H12B	0.458347	0.141763	0.687737	0.039*	
C13	0.72017 (19)	0.07935 (11)	0.63497 (11)	0.0367 (3)	
H13A	0.654624	−0.016172	0.615835	0.044*	
H13B	0.699122	0.094119	0.553654	0.044*	
C14	0.9572 (2)	0.11511 (13)	0.69000 (12)	0.0434 (3)	
H14A	0.978706	0.092272	0.766768	0.052*	
H14B	1.025346	0.062640	0.625831	0.052*	
C15	1.06093 (18)	0.26264 (12)	0.72628 (12)	0.0395 (3)	
H15A	1.049767	0.283741	0.648142	0.047*	
H15B	1.213989	0.284207	0.764521	0.047*	
C16	0.95595 (16)	0.34778 (10)	0.82147 (10)	0.0289 (2)	
H16A	1.022076	0.443016	0.839636	0.035*	
H16B	0.979598	0.333547	0.902950	0.035*	
C17	0.62132 (16)	0.39617 (10)	0.87219 (10)	0.0271 (2)	
C21	0.07710 (15)	0.31985 (9)	0.34127 (9)	0.0248 (2)	
C22	−0.12496 (17)	0.22400 (10)	0.34711 (11)	0.0307 (2)	
H22A	−0.088458	0.147749	0.361614	0.037*	
H22B	−0.183688	0.270495	0.420145	0.037*	
C23	−0.29341 (19)	0.17261 (13)	0.22306 (13)	0.0436 (3)	
H23A	−0.417294	0.106103	0.227129	0.052*	
H23B	−0.343043	0.247436	0.214122	0.052*	
C24	−0.2051 (3)	0.10925 (14)	0.10684 (13)	0.0548 (4)	
H24A	−0.171321	0.028068	0.110666	0.066*	
H24B	−0.314381	0.082553	0.027866	0.066*	
C25	−0.0056 (2)	0.20478 (15)	0.10149 (11)	0.0479 (3)	
H25A	−0.042502	0.281655	0.088612	0.057*	
H25B	0.052018	0.159214	0.027509	0.057*	
C26	0.16431 (19)	0.25464 (12)	0.22421 (10)	0.0342 (2)	
H26A	0.289135	0.320050	0.219506	0.041*	
H26B	0.211744	0.179045	0.232897	0.041*	
C27	0.24348 (16)	0.36510 (10)	0.46538 (10)	0.0262 (2)	

### 1-Hydroxycyclohexanecarboxylic acid Atomic displacement parameters (Å^2^)

**Table:**

	*U*^11^	*U*^22^	*U*^33^	*U*^12^	*U*^13^	*U*^23^
O11	0.0288 (4)	0.0280 (4)	0.0239 (3)	0.0088 (3)	0.0008 (3)	0.0105 (3)
O12	0.0645 (6)	0.0432 (5)	0.0353 (4)	0.0300 (4)	0.0260 (4)	0.0205 (4)
O13	0.0612 (6)	0.0326 (4)	0.0472 (5)	0.0244 (4)	0.0300 (4)	0.0201 (4)
O21	0.0329 (4)	0.0295 (4)	0.0428 (4)	0.0166 (3)	0.0127 (3)	0.0213 (3)
O22	0.0386 (4)	0.0302 (4)	0.0330 (4)	0.0165 (3)	−0.0036 (3)	0.0091 (3)
O23	0.0368 (4)	0.0323 (4)	0.0367 (4)	0.0150 (3)	0.0054 (3)	0.0043 (3)
C11	0.0254 (5)	0.0236 (4)	0.0234 (4)	0.0080 (4)	0.0048 (3)	0.0094 (4)
C12	0.0344 (5)	0.0240 (5)	0.0381 (6)	0.0063 (4)	0.0111 (4)	0.0102 (4)
C13	0.0450 (6)	0.0248 (5)	0.0380 (6)	0.0120 (4)	0.0104 (5)	0.0071 (4)
C14	0.0494 (7)	0.0436 (7)	0.0438 (7)	0.0299 (6)	0.0105 (5)	0.0137 (5)
C15	0.0254 (5)	0.0487 (7)	0.0430 (6)	0.0157 (5)	0.0060 (5)	0.0125 (5)
C16	0.0268 (5)	0.0318 (5)	0.0261 (5)	0.0084 (4)	−0.0001 (4)	0.0103 (4)
C17	0.0279 (5)	0.0253 (5)	0.0282 (5)	0.0077 (4)	0.0073 (4)	0.0097 (4)
C21	0.0275 (5)	0.0247 (4)	0.0285 (5)	0.0127 (4)	0.0081 (4)	0.0137 (4)
C22	0.0305 (5)	0.0288 (5)	0.0368 (5)	0.0096 (4)	0.0084 (4)	0.0160 (4)
C23	0.0322 (6)	0.0398 (6)	0.0527 (7)	0.0053 (5)	−0.0029 (5)	0.0171 (6)
C24	0.0618 (9)	0.0474 (7)	0.0398 (7)	0.0174 (7)	−0.0119 (6)	0.0041 (6)
C25	0.0646 (9)	0.0616 (8)	0.0273 (6)	0.0371 (7)	0.0111 (5)	0.0161 (5)
C26	0.0397 (6)	0.0432 (6)	0.0321 (5)	0.0245 (5)	0.0153 (4)	0.0187 (5)
C27	0.0255 (5)	0.0263 (5)	0.0308 (5)	0.0101 (4)	0.0092 (4)	0.0128 (4)

### 1-Hydroxycyclohexanecarboxylic acid Geometric parameters (Å, º)

**Table:**

O11—C11	1.4223 (11)	C15—C16	1.5234 (15)
O11—H11	0.8400	C15—H15A	0.9900
O12—C17	1.3030 (13)	C15—H15B	0.9900
O12—H12	0.8400	C16—H16A	0.9900
O13—C17	1.2221 (12)	C16—H16B	0.9900
O21—C21	1.4280 (11)	C21—C26	1.5290 (14)
O21—H21	0.8400	C21—C27	1.5306 (14)
O22—C27	1.3206 (12)	C21—C22	1.5339 (14)
O22—H22	0.8400	C22—C23	1.5293 (16)
O23—C27	1.2114 (12)	C22—H22A	0.9900
C11—C17	1.5285 (13)	C22—H22B	0.9900
C11—C12	1.5321 (14)	C23—C24	1.521 (2)
C11—C16	1.5369 (13)	C23—H23A	0.9900
C12—C13	1.5282 (15)	C23—H23B	0.9900
C12—H12A	0.9900	C24—C25	1.518 (2)
C12—H12B	0.9900	C24—H24A	0.9900
C13—C14	1.5197 (17)	C24—H24B	0.9900
C13—H13A	0.9900	C25—C26	1.5256 (17)
C13—H13B	0.9900	C25—H25A	0.9900
C14—C15	1.5187 (18)	C25—H25B	0.9900
C14—H14A	0.9900	C26—H26A	0.9900
C14—H14B	0.9900	C26—H26B	0.9900
			
C11—O11—H11	109.5	O13—C17—C11	121.55 (9)
C17—O12—H12	109.5	O12—C17—C11	114.61 (8)
C21—O21—H21	109.5	O21—C21—C26	106.88 (8)
C27—O22—H22	109.5	O21—C21—C27	108.30 (8)
O11—C11—C17	108.93 (7)	C26—C21—C27	111.13 (8)
O11—C11—C12	107.19 (8)	O21—C21—C22	110.07 (8)
C17—C11—C12	111.64 (8)	C26—C21—C22	110.98 (9)
O11—C11—C16	110.56 (8)	C27—C21—C22	109.42 (8)
C17—C11—C16	107.46 (8)	C23—C22—C21	111.25 (9)
C12—C11—C16	111.07 (8)	C23—C22—H22A	109.4
C13—C12—C11	111.45 (9)	C21—C22—H22A	109.4
C13—C12—H12A	109.3	C23—C22—H22B	109.4
C11—C12—H12A	109.3	C21—C22—H22B	109.4
C13—C12—H12B	109.3	H22A—C22—H22B	108.0
C11—C12—H12B	109.3	C24—C23—C22	111.30 (10)
H12A—C12—H12B	108.0	C24—C23—H23A	109.4
C14—C13—C12	111.29 (9)	C22—C23—H23A	109.4
C14—C13—H13A	109.4	C24—C23—H23B	109.4
C12—C13—H13A	109.4	C22—C23—H23B	109.4
C14—C13—H13B	109.4	H23A—C23—H23B	108.0
C12—C13—H13B	109.4	C25—C24—C23	111.29 (11)
H13A—C13—H13B	108.0	C25—C24—H24A	109.4
C15—C14—C13	110.73 (9)	C23—C24—H24A	109.4
C15—C14—H14A	109.5	C25—C24—H24B	109.4
C13—C14—H14A	109.5	C23—C24—H24B	109.4
C15—C14—H14B	109.5	H24A—C24—H24B	108.0
C13—C14—H14B	109.5	C24—C25—C26	111.49 (11)
H14A—C14—H14B	108.1	C24—C25—H25A	109.3
C14—C15—C16	111.48 (10)	C26—C25—H25A	109.3
C14—C15—H15A	109.3	C24—C25—H25B	109.3
C16—C15—H15A	109.3	C26—C25—H25B	109.3
C14—C15—H15B	109.3	H25A—C25—H25B	108.0
C16—C15—H15B	109.3	C25—C26—C21	110.72 (9)
H15A—C15—H15B	108.0	C25—C26—H26A	109.5
C15—C16—C11	110.93 (8)	C21—C26—H26A	109.5
C15—C16—H16A	109.5	C25—C26—H26B	109.5
C11—C16—H16A	109.5	C21—C26—H26B	109.5
C15—C16—H16B	109.5	H26A—C26—H26B	108.1
C11—C16—H16B	109.5	O23—C27—O22	123.50 (9)
H16A—C16—H16B	108.0	O23—C27—C21	123.62 (9)
O13—C17—O12	123.82 (9)	O22—C27—C21	112.86 (8)
			
O11—C11—C12—C13	66.73 (11)	O21—C21—C22—C23	62.91 (11)
C17—C11—C12—C13	−174.05 (9)	C26—C21—C22—C23	−55.19 (11)
C16—C11—C12—C13	−54.14 (12)	C27—C21—C22—C23	−178.18 (8)
C11—C12—C13—C14	55.28 (13)	C21—C22—C23—C24	54.87 (13)
C12—C13—C14—C15	−56.39 (13)	C22—C23—C24—C25	−55.24 (14)
C13—C14—C15—C16	57.12 (13)	C23—C24—C25—C26	56.14 (14)
C14—C15—C16—C11	−56.21 (12)	C24—C25—C26—C21	−56.31 (13)
O11—C11—C16—C15	−64.41 (11)	O21—C21—C26—C25	−64.35 (12)
C17—C11—C16—C15	176.83 (9)	C27—C21—C26—C25	177.67 (9)
C12—C11—C16—C15	54.46 (11)	C22—C21—C26—C25	55.67 (12)
O11—C11—C17—O13	−21.31 (13)	O21—C21—C27—O23	19.06 (13)
C12—C11—C17—O13	−139.49 (10)	C26—C21—C27—O23	136.17 (11)
C16—C11—C17—O13	98.50 (11)	C22—C21—C27—O23	−100.93 (11)
O11—C11—C17—O12	160.57 (9)	O21—C21—C27—O22	−162.34 (8)
C12—C11—C17—O12	42.39 (12)	C26—C21—C27—O22	−45.24 (12)
C16—C11—C17—O12	−79.62 (11)	C22—C21—C27—O22	77.66 (10)

### 1-Hydroxycyclohexanecarboxylic acid Hydrogen-bond geometry (Å, º)

**Table:**

*D*—H···*A*	*D*—H	H···*A*	*D*···*A*	*D*—H···*A*
O11—H11···O21^i^	0.84	1.89	2.7195 (10)	169
O12—H12···O13^ii^	0.84	1.80	2.6359 (11)	176
O21—H21···O23^iii^	0.84	1.95	2.7716 (11)	166
O22—H22···O11	0.84	1.84	2.6594 (10)	164
C26—H26*A*···O13^i^	0.99	2.58	3.4349 (14)	145

Symmetry codes: (i) −*x*+1, −*y*+1, −*z*+1; (ii) −*x*+1, −*y*+1, −*z*+2; (iii) −*x*, −*y*+1, −*z*+1.
